# Development of an implementation intervention to promote adoption of the COMFORT clinical practice guideline for peripartum pain management: a qualitative study

**DOI:** 10.1186/s43058-024-00687-5

**Published:** 2025-01-02

**Authors:** Limi Sharif, Shelytia Cocroft, Shawna N. Smith, Christopher Benincasa, Alex F. Peahl, Lisa Kane Low, Jennifer Waljee, Carrie Miller, Carey Simpson, Michelle H. Moniz

**Affiliations:** 1 Department of Anesthesiology, University of Michigan, Ann Arbor, USA; 2 Department of Obstetrics and Gynecology, University of Michigan, 2800 Plymouth Rd., Building #10, Rm G016, Ann Arbor, MI 48109-5276 USA; 3 Department of Health Management & Policy, School of Public Health, University of Michigan, Ann Arbor, USA; 4 Department of Psychiatry, University of Michigan, Ann Arbor, USA; 5 Obstetrics Initiative, Ann Arbor, USA; 6 University of Michigan Institute for Healthcare Policy and Innovation, Ann Arbor, USA; 7 Department of Learning Health Sciences, University of Michigan Medical School, Ann Arbor, USA; 8 University of Michigan School of Nursing, Ann Arbor, USA; 9 Center for Healthcare Outcomes and Policy (CHOP), Ann Arbor, USA; 10 Michigan Opioid Prescribing Network, Ann Arbor, USA; 11 Department of Surgery, University of Michigan, Ann Arbor, USA

**Keywords:** Pain, Peripartum, Implementation, Guideline, Maternity care, Intervention, CFIR, E-REP, ERIC

## Abstract

**Background:**

Pain management after childbirth is widely variable, increasing risk of untreated pain, opioid harms, and inequitable experiences of care. The Creating Optimal Pain Management FOR Tailoring Care (COMFORT) clinical practice guideline (CPG) seeks to promote evidence-based, equitable acute peripartum pain management in the United States. We aimed to identify contextual conditions (i.e., barriers and facilitators) and discrete implementation strategies (i.e., theory-based actions taken to routinize a clinical practice) likely to influence COMFORT CPG uptake and specify corresponding multi-component implementation interventions at the perinatal quality collaborative- and unit-level.

**Methods:**

We conducted a qualitative study involving virtual individual interviews and focus groups. Interviews included individuals undergoing childbirth from 2018–2023, (recruited through two online registries), and actively practicing maternity clinicians and surgeons, (recruited via snowball sampling with the eDelphi panel creating the COMFORT CPG), caring for pregnant people in the United States. Focus groups included physicians, midwives, nurses, and unit-based quality improvement (QI) staff working at Michigan hospitals within the Obstetrics Initiative, a statewide perinatal quality collaborative funded by Blue Cross Blue Shield of Michigan and Blue Care Network. The Consolidated Framework for Implementation Research, Expert Recommendations for Implementing Change taxonomy, and Replicating Effective Programs framework informed data collection and analysis. Qualitative content analysis characterized influential contextual conditions, which were linked to implementation strategies and tools using principles of implementation mapping. We then specified multi-component implementation interventions for use by quality collaboratives and unit-based teams.

**Results:**

From May–September 2023, we completed 57 semi-structured individual interviews (31 patients, 26 clinicians) and six focus groups (44 QI champions). Participants identified 10 key conditions influential for COMFORT CPG adoption. Findings enabled identification of five collaborative-level implementation strategies, 27 unit-level implementation strategies, and 12 associated tools to promote COMFORT CPG adoption including the specification of each strategy’s hypothesized mechanism of action and each tool’s goal and potential uses.

**Conclusions:**

This work identifies contextual conditions and implementation strategies and tools at the perinatal quality collaborative and unit levels to promote COMFORT CPG adoption on maternity units. These findings may foster more rapid CPG implementation and thereby promote more equitable and evidence-based perinatal pain management care.

Contributions to the literature

- The Creating Optimal pain Management FOR Tailoring care (COMFORT) guideline promotes equitable, evidence-based pain management experiences for birthing people.
- Using the Consolidated Framework for Implementation Research, the Expert Recommendations for Implementing Change taxonomy, the Replicating Effective Programs framework, and Implementation Mapping, we identified influential contextual conditions and corresponding implementation strategies and tools with theorized mechanisms of action on COMFORT guideline implementation on maternity units in the United States.
- Quality improvement leaders can utilize the implementation resources herein to help promote uptake of evidence-based practices for pain management after childbirth.

## Background

Inadequate pain management after childbirth increases individuals’ risks of delayed recovery [1], breastfeeding cessation [2], peripartum mood disorders [3], poor parent-infant bonding [4], and development of chronic pain [5]. While opioids are an effective tool for acute pain management, excess opioid prescribing incurs risk of persistent use [6, 7], increased healthcare costs and utilization [8], diversion to unintended users [9], overdose [10], and adverse health outcomes in infants [11]. Postpartum opioid prescribing is widely variable, leaving patients at risk of both insufficient pain control and harms from opioid overuse [6, 9, 12–27]. The peripartum period is also fraught with significant inequities in pain management experiences, with people of color and other marginalized individuals more likely to experience severe pain following cesarean births [28], fewer postprocedural pain assessments [29], and dismissal of concerns [30]. To address these gaps, our team conducted a systematic review and led an eDelphi consensus process, informed by the RAND-UCLA Appropriateness Methodology, to develop the Creating Optimal pain Management FOR Tailoring care (COMFORT) clinical practice guideline (CPG).

However, the impact of a CPG depends not only on its effectiveness, but also the extent to which it is properly implemented and reaches desired populations. Unfortunately, clinical practice guidelines regularly go unused [31], even after active implementation efforts [32–34].

Perinatal quality collaboratives, (or others leading multi-site, shared quality improvement [QI] efforts, such as healthcare system quality departments or statewide departments of health), may play an important role in facilitating implementation of COMFORT-recommended evidence-based practices. Quality collaboratives often generate toolkits (i.e., packages of standard, scalable resources) and create group-based learning opportunities (e.g., webinars, workgroups), or offer technical assistance to support unit-based QI teams in promoting clinical behavior change within their organizations [35]. However, when a perinatal quality collaborative successfully changes practice, results are often not reproducible because strategies used are inconsistently labeled, inadequately described, rarely justified theoretically, or grouped into implementation “bundles” whose specific elements are poorly understood or evaluated [36]. Formative work to guide the design of collaborative-wide resources and activities may enhance their acceptability, utility, and effectiveness [37]. Moreover, like any intervention, more precise description of collaborative-level resources and activities and their proposed mechanisms of action [38–42] may enable their measurement, evaluation, and reproducibility—ultimately fostering more effective maternity QI efforts at scale.

Additionally, prior work on clinical practice change in healthcare settings suggests that unit-based teams can execute specific improvement strategies (e.g., electronic health record [EHR] updates, clinician education) that optimize organizational contextual conditions (e.g., clinician attitudes toward a CPG, unit protocols, staffing models, employee teamwork), and thereby promote ongoing uptake of CPGs [43, 44]. Formative work to characterize likely organizational barriers and facilitators to CPG uptake—and promising corresponding improvement strategies and tools that unit-based QI teams can utilize—could foster more effective efforts to promote COMFORT CPG uptake in the United States. A priori specification of these strategies and tools also enables prospective tracking of their use by hospital-based QI teams and their associated effects on implementation and clinical outcomes.

Therefore, to inform effective large-scale adoption of the COMFORT CPG in the United States, we conducted a qualitative study with three objectives: 1) characterize contextual conditions that could influence COMFORT CPG adoption on maternity units, 2) identify corresponding implementation strategies and tools with plausible mechanisms to optimize conditions and promote desired implementation and downstream clinical outcomes, and 3) specify implementation interventions that unit-based teams and perinatal quality collaboratives can deploy to embed the COMFORT CPG in routine clinical practice.

## Methods

In preparation for publication of the 2025 COMFORT CPG, we initiated work to prepare for CPG implementation across maternity units and perinatal quality collaboratives in the United States. Specifically, we conducted a qualitative study involving interviews and focus groups with maternity patients and clinicians in the United States, with a goal of identifying key contextual conditions, strategies, and tools likely to influence COMFORT CPG implementation in the United States. We report our methods according to the Consolidated Criteria for Reporting Qualitative Research (COREQ) [45] because of its detailed and specific focus on the collection, analysis, and reporting of interview data, such as that used in the current study. The completed checklist is available in Additional file 1.

### Clinical innovation: the COMFORT Clinical Practice Guideline

The COMFORT CPG was developed through generation of two systematic reviews and conduct of an eDelphi panel that generated clinical practice recommendations for peripartum pain management. The eDelphi panel included maternity care physicians, pain specialists, and patient advocates. Using a consensus process informed by the RAND/UCLA Appropriateness Method [46], the panel developed recommendations in six key areas to help clinicians promote equitable, evidence-based pain management for patients after childbirth: 1) robust counseling and education about pain expectations and management options; 2) use of scheduled acetaminophen and ibuprofen as first-line for pain; 3) tailored opioid prescribing as needed through shared decision-making; 4) supplementation of medications with non-pharmacologic strategies to augment pain management; 5) incorporation of inpatient pain management options (e.g., regional anesthesia, patient-controlled analgesia); and 6) specific recommendations for patients with complex pain needs (e.g., history of opioid use disorder, chronic pain).

### Participants and Data Collection

We utilized individual semi-structured interviews to elicit patient- and clinician-level barriers and facilitators to CPG adoption and potential strategies and tools to promote CPG adoption. Focus groups then confirmed patient- and clinician-level determinants, newly assessed organizational-level barriers and facilitators to CPG adoption within a statewide quality collaborative, and solicited participant input on draft implementation tools planned for collaborative-wide distribution.

Interviewees included a national convenience sample of individuals who underwent childbirth in the United States from 2018–2023 and maternity care clinicians (e.g., physicians, nurses, midwives) with active practices in the United States. Birthing people were recruited through two online registries (UMResearch.org, ResearchMatch.org). Clinicians were recruited via snowball sampling based on referrals from the eDelphi panel that generated the COMFORT CPG, and largely included clinicians with specialized expertise in pain management, substance use disorder, and/or care of marginalized populations. From May 2023-July 2023, we conducted virtual semi-structured interviews lasting approximately 60 min, led by LS and SC. Interviews were recorded with permission and professionally transcribed.

For focus groups, we recruited a convenience sample of QI “champions” (i.e., the physicians, midwives, nurses, and data abstractors leading QI efforts) working in Michigan hospitals that participate in the Obstetrics Initiative (OBI), a statewide quality collaborative funded by Blue Cross Blue Shield of Michigan (BCBSM) and Blue Care Network as part of the BCBSM Value Partnerships Program [47]. Although BCBSM and OBI work collaboratively, the opinions, beliefs, and viewpoints expressed by the authors do not necessarily reflect the opinions, beliefs, and viewpoints of BCBSM or any of its employees.

Based on their role within OBI member hospitals, these individuals have unique knowledge about implementation of clinical guidelines and are ideally positioned to share insights about how various unit conditions might affect guideline adoption. From August–September 2023, SC and MHM facilitated virtual focus groups, each with 4–10 participants and lasting approximately 90 min. In addition to assessing perceived barriers, facilitators, and promising implementation strategies, focus groups asked unit-based QI champions about prototype resources for a collaborative-wide implementation toolkit. Based on interview findings, MHM and AP generated a draft list of tools and content for each. These draft tools were shared during focus groups to elicit desires for tool additions and refinements. Focus groups were recorded with permission and transcribed using Firefly software.

Field notes were taken during interviews and focus groups and used to develop memos reviewed during analysis.

### Theoretical framework

**Fig. 1:**
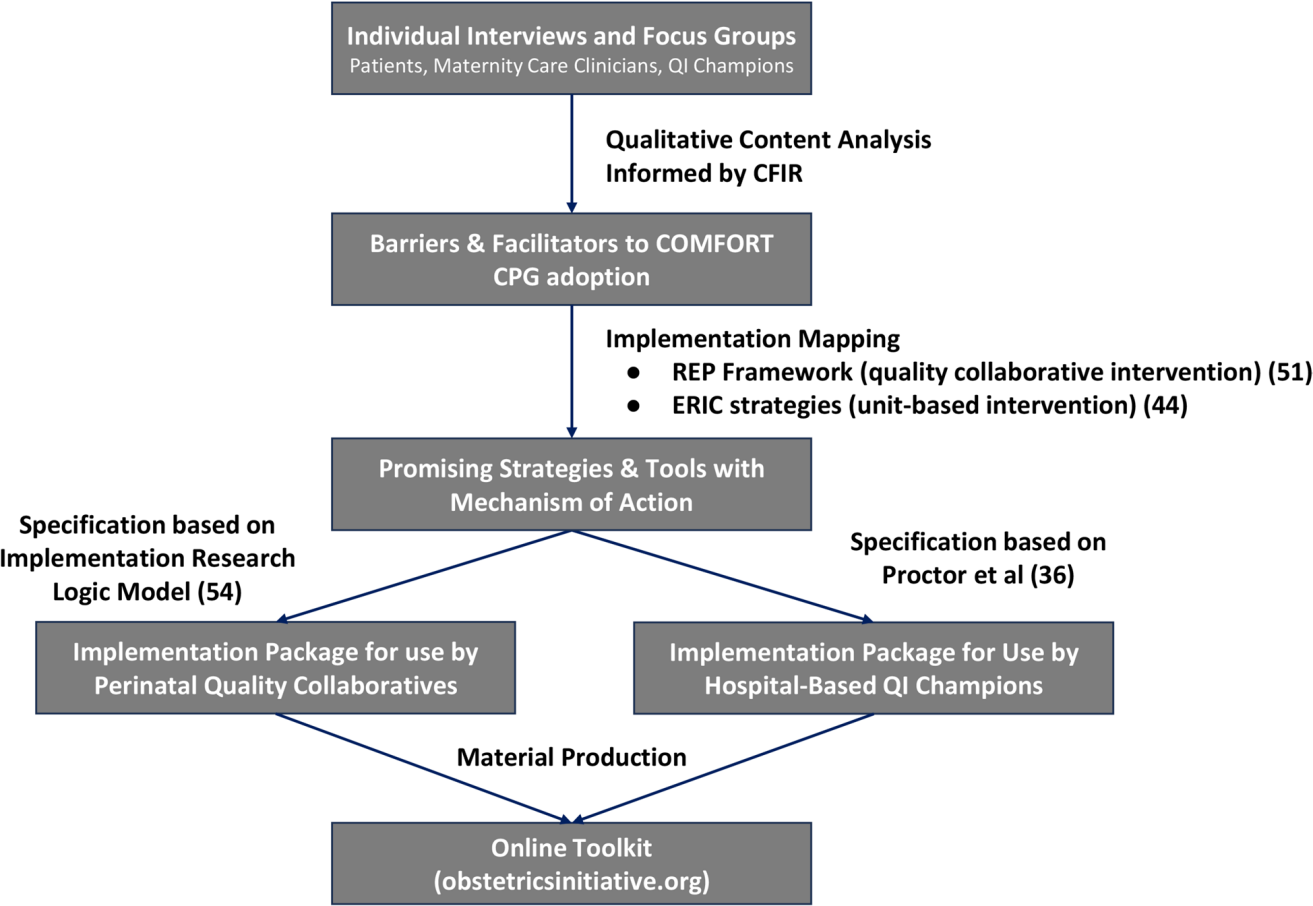
Flowchart of Methods and Deliverables

Implementation science determinant frameworks provide a systematic approach to identify key barriers and facilitators to changing individual and organizational behavior and increase the probability of replicable improvements in practice [48, 49]. One such determinant framework, the Consolidated Framework for Implementation Research (CFIR), guided this study a priori, informing data collection (i.e., semi-structured interview and focus group guides) and analyses (i.e., coding framework). CFIR includes 39 contextual conditions in five domains (Clinical Innovation, Outer Setting, Inner Setting, Individuals, Implementation Process) that can contain barriers and facilitators that may influence implementation of an evidence-based practice [50]. Our interview and focus group guides and analyses were also informed by the Enhanced Replicating Effective Programs framework (EREP; an implementation package with five core strategies) and the Expert Recommendations for Implementing Change (ERIC; a taxonomy of 73 discrete implementation strategies), which were used to identify promising strategies and tools with theorized mechanisms of optimizing key CFIR-based conditions [44, 51]. The interview and focus group guides contained probes about select CFIR constructs and specific EREP and ERIC strategies, which were refined via pilot testing with two birthing people and two obstetricians. Pilot participants provided feedback about CFIR constructs and strategies thought less likely to be relevant to postpartum pain management, guiding the authors’ development of a more parsimonious interview guide. Nearly all interviews were completed and analyzed prior to conducting focus groups, and preliminary interview findings informed development of the focus group guide. The codebook retained all CFIR constructs, EREP components, and ERIC strategies to allow all potentially relevant themes and relationships to emerge from the data during analysis. Our research team utilized principles of Implementation Mapping to make clear, theoretical linkages between identified CFIR determinants and strategies and tools via theorized mechanisms on implementation (proximal) outcomes and clinical (downstream/distal) outcomes [52–54]. Via this process, we identified strategies/tools with hypothesized mechanisms of action on determinants likely to affect implementation outcomes. We then specified theory-based implementation interventions for perinatal quality collaboratives using Proctor et al.’s guidance [36], and for unit-based QI teams using the Implementation Research Logic Model [55] (Fig. 1).

### Data analysis

Data analysis steps were informed by the implementation mapping process. We used iterative, consensus coding throughout, with rare discrepancies resolved via discussions to reach consensus. LS, SC, and MHM led the coding process using Dedoose software version 9.0.90 (interviews and focus groups) and Excel spreadsheets (mapping CFIR determinants to ERIC strategies, EREP components, and tools via hypothesized mechanisms of action).

First, we conducted a qualitative content analysis, applying CFIR constructs within the Clinical Innovation, Outer Setting, Inner Setting, and Individuals domains as a priori codes to interview and focus group data to understand potential barriers and facilitators to CPG implementation. We synthesized all interview and focus group data relevant to each CFIR construct and compared across constructs to identify and characterize the conditions with strongest anticipated influence on implementation outcomes. We developed matrices to link CFIR conditions to potentially helpful strategies and tools emerging in the data.

Second, we identified strategies and tools with plausible mechanisms for optimizing these CFIR conditions and thereby enhancing implementation and clinical outcomes. Using principles of Implementation Mapping, the research team articulated desired mechanisms to optimize influential CFIR conditions. We then identified strategies and associated tools that could plausibly act via these mechanisms by building matrices linking each CFIR condition to strategies and tools explicitly described by interview and focus group participants as helpful to address barriers. We then added strategies and associated tools, identified by SC and MHM, via a manual comparison of the five EREP components and the 73 ERIC strategies to identified CFIR conditions and associated qualitative themes. From this list, the research team only retained strategies with anticipated universal utility and feasibility across diverse maternity settings and tools with anticipated universal utility that the research team could feasibly generate.

Third, to specify an implementation package for quality collaboratives to provide to unit-based teams, we described each component of the EREP framework [i.e., 1) User-friendly “packaging” of the CPG; 2) Structured provider training; 3) Performance feedback; 4) Technical assistance; and 5) Facilitation], as discrete implementation strategies. As recommended by Proctor et al. [36], we included each strategy’s hypothesized mechanism of action on specific implementation outcomes [56], with a goal of enabling future prospective evaluation of this intervention. Given that OBI offers a Pay for Performance (P4P) financial incentive to member hospitals, we also used end-user input via our qualitative data to specify a performance metric designed to incentivize COMFORT-concordant clinical practices as part of our statewide implementation intervention. Then, we generated a table with each tool’s goal, CFIR condition addressed, and potential uses. OBI staff, in partnership with MHM, then created all core tools using this study’s findings to inform specific content creation. To specify the implementation intervention for unit-based teams, SC and MHM generated a logic model mapping CFIR determinants to unit-based teams’ strategies and associated tools, with hypothesized mechanisms of action on implementation outcomes. Strategies were categorized into the four CFIR Implementation Process domain constructs (Teaming, Planning, Engaging, Reflecting & Evaluating), based on OBI’s standard approach for communicating core strategies to member hospitals.

Findings were member-checked with interview and focus group participants by email. Our research team consisted of research assistants (LS, CB); clinicians with expertise in obstetrics (AP, LKL, MHM); experts in qualitative interviewing and analysis (SC, AP, LKL, MHM); and specialists in implementation science, intervention planning, and implementation process analysis (SS, JW).

## Results

We completed 53 semi-structured interviews (31 patients and 26 clinicians; mean interview duration, 60 min, range 55–70 min) and six 90-min focus groups (44 maternity care QI champions from 35 hospitals; Table 1, 2) with key informants. Our analysis suggests that COMFORT CPG implementation will be influenced by complex interactions between contextual conditions, the “meta” strategies used by perinatal quality collaboratives to support unit-based QI teams, and the strategies and tools deployed by unit-based QI teams to optimize local contextual conditions.

**Table 1:** Individual interview participant demographics

Characteristic	n (%)
*Patients (N* = *31)*	
Race/Ethnicity	
Black/African American	13 (42)
White	10 (32)
Hispanic	2 (6)
Asian	1 (3)
American Indian/Alaska Native	2 (6)
Multiracial	3 (10)
Geographic Region	
Northeast	13 (42)
Midwest	7 (23)
Southeast	5 (15)
West	3 (10)
Southwest	3 (10)
*Clinicians (N* = *26)*	
Race/Ethnicity	
Black/African American	4 (15)
White	18 (77)
Hispanic	0 (0)
Asian	3 (12)
American Indian/Alaska Native	1 (4)
Multiracial	0 (0)
Geographic Region	
Northeast	5 (19)
Midwest	10 (38)
Southeast	5 (19)
West	5 (19)
Southwest	1 (4)
Clinician Type^a^	
OB/GYN	9 (35)
Family Medicine	2 (8)
Pain Medicine	2 (8)
Addiction Medicine	3 (11)
Maternal Fetal Medicine	2 (8)
Surgeon	6 (23)
Certified Nurse Midwife/Certified Midwife	3 (11)
Perinatal Registered Nurse	1 (4)

^a^Participants could select more than one clinician type

**Table 2:** Role of quality improvement champions participating in focus groups with OBI hospitals

Champion Role	n (%)
Physician	8 (18)
Nurse	17 (39)
Midwife	2 (5)
Clinical Data Abstractor	14 (32)
Quality and Safety Champion	3 (7)

*OBI* Obstetrics Initiative

### Contextual conditions

In our CFIR-based analysis of contextual conditions, 10 conditions emerged as important to optimize COMFORT CPG adoption, shown with illustrative qualitative data in Table 3.

**Table 3:** Key Conditions for COMFORT CPG Implementation

Innovation Conditions	
Characteristics of COMFORT CPG	"I don't think anybody's really going to question this particular kind of recommendation or this kind of the evidence based surrounding this…some of us have already made some of these changes nationally. There's just a big push for less opioids… So I don't think people are going to be questioning whether or not the evidence is there to make some of these changes." ***(Clinician, Family Physician)*** “I'm hoping too that using a clinical practice guideline and standardizing will help with some of the implicit bias and the inequities in pain control with our patients of color. And by standardizing that we'll start to see improvements in care for our patients that are not receiving the same care…” (***Clinician, MFM***)
Outer Setting Conditions	
Standard of Care	“I fully support nationalized protocols and guidelines to try to make the standard of care across all hospitals.…But I also think it's really busy in obstetric care, and unless something is nationalized…it may not be readily adopted by obstetricians without that requirement.” ***(Clinician, MFM)*** ***“***'It’s a lot easier to get people on board if you have some precise guidelines and you can say this is the guidelines that everyone in the state is going to be using.” ***(Clinician, OBGYN)***
Inner Setting Conditions	
Relative Priority	“[W]e have a ton of projects going on in the OB department specifically, and there's going to be hesitation at first because this is just one more thing” ***(Quality Champion)*** “…the conflicting projects is what leads the hesitation, I think… but if it's what's best for the patient, I mean, we're going to do it… [if] it's evidence based, this is what we should be doing.” ***(Clinician, Nurse)***
Structural Characteristics	“It has to be done on a system wide level, not just like an individual site level. And that's why it takes so long, because they literally have to go to every site and get approval to change an order set… it's not just as easy as you would think it would be of implementing new order set. It's going to be a really lengthy process for us.” ***(Clinical Data Abstractor)*** “We run into a lot of barriers there, especially in a small community hospital, getting those resources. I mean, we can put an abdominal binder on, but pretty much past that, I don't think we have many or any of those other multimodal factors.” ***(Clinician, Family Physician)***
Workflow Compatibility	“When I'm in a resident clinic with… we had 40 some patients for me and a resident to see the other day that all have multiple other high risk conditions. They've got uncontrolled diabetes or hypertension that we're trying to manage or some other medical problem. Pain control unfortunately is not on top of my priority. We're kind of lucky if we get some of the basics done just to make sure that we at least have a birth control plan and that we've talked about pain management in labor, if we've not even gotten that far.” ***(Clinician, OBGYN)*** “I think everybody's going to be like, yes, these are good things. We should be talking about them. But if you put a little microphone in the corner of the room…it's going to get forgotten a lot of times, or it's going to get pushed past, or it's going to take a long time to become part of what I talk about. ***(Clinician, Family Physician)*** “A lot of our providers are private practice and full offices, busy offices, and aren't wanting phone calls…And to kind of avoid that, a lot of them are giving a larger number of opioids on discharge.” ***(Clinician, Family Physician)*** “So I think just figuring out how to have that conversation with the patients and who's going to be in charge of having that conversation will be kind of the struggle for us…I think just historically with changes and adding more things onto providers’ plates, it becomes a difficulty. Is it the nurse's responsibility or the physician's responsibility to have that conversation?” ***(Clinician, Nurse)***
Culture of Learning	“Our institution, usually we have a pushback for a good three months, and then the second set of three months, they're like, okay, we can kind of see what you're saying. And usually by year's end, we have a really good uptake of evidence based changes like this.” ***(Clinical Data Abstractor)*** “I struggle a little bit with standardized care, with my providers, with some of the groups. We have groups rather than hospital physicians, and they, in general, struggle with cookie cutter practice and feel as though they like to follow their gut in many circumstances.” ***(Quality and Safety Champion)***
Individual Conditions	
QI Team Characteristics	"[W]e …need to start with the why… lead with why are we doing this?" ***(Quality and Safety Champion)*** "You just need to get a couple of key people to be on board with it and they can bring everyone else along…if you have a couple of people who are enthusiastic about it and are talking it up, it makes all the difference." ***(Clinician, OBGYN)***
Provider Characteristics	“I'm really excited to see that there's a tool to individualize those [opioid] prescriptions more. I think that it'll be great from an education standpoint and teaching the residents too. And I don't think that there's going to be a lot of resistance with our physicians here” ***(Clinician, MFM)*** “I don't think [physicians] like to be told what to do. I mean, even if you said this is the national guidelines… most of them know it, and they still kind of do what they want to do.” ***(Clinician, Nurse)*** "I think this is where evidence base is really going to matter. If you're going to tell me the proverbial me, ‘Give this patient five norco instead of ten,’ I want to know why I'm going to be giving them five instead of ten if I've been prescribing ten to my C-section patients for 20 years." ***(Clinician, Family Physician)***
Nurse Characteristics	“I do find a couple of the nurses that kind of think it's silly that we do [scheduled Tylenol and Motrin]. They really think the patient should request… They don't have that conversation and they'll let the patient refuse these scheduled meds. But then you'll look back at their chart and they're taking Oxycodone but haven't been taking Motrin and Tylenol.” ***(Clinician, Nurse)*** “When I worked at the bedside, I could definitely see a difference when I staggered my medications versus together.” ***(Clinician, Nurse)***
Patient Characteristics	“So I think the patient education to support shared decision making will be really big…It'll be a part of the process. And so people are going to engage patients and ask questions, and patients will be able to answer and go from there. And so I think that's great for people who maybe haven't had a voice previously…” ***(Patient)*** “We set up a follow-up visit and we hope for the best… relying on patients to reach back out if things aren't well controlled… At four weeks postpartum, we hear people say, ‘Oh my gosh, I was just dying. I was hurting so bad, I just didn't know what to do.’ And it's like, ‘Oh, sorry. We don't have a system set up for that.’” ***(Clinician, Midwife)*** “…[E]ven over the counter, if you are really low on money, you're probably going to pick the formula or diapers or wipes before you pick your right [sic] pain meds.” ***(Patient)***

*CPG* clinical practice guideline, *MFM* maternal fetal medicine, *OBGYN* obstetrician-gynecologist

#### Characteristics of the COMFORT CPG

Many key informants expressed positive reactions to the CPG, noting perceived robustness of the CPG development process, credibility and trustworthiness of the professional societies endorsing the CPG, and strength of the evidence underlying the recommendations. Key informants also noted how the CPG had advantages over current practice. Specifically, they recognized the CPG as a promising intervention to promote better opioid stewardship after childbirth. Informants appreciated the CPG’s call for personalization in care plan development, including shared-decision making during pre-procedural counseling and tailored opioid prescribing within the CPG ranges. Additionally, clinicians described valuing the specificity and clarity of the opioid prescribing ranges stratified by the patient’s history and procedure type and noted how this could better standardize prescribing practices. Many noted that the standardization offered by the CPG could address existing inequities in pain management care by promoting provision of anticipatory counseling about pain management and evidence-based treatment for all patients.

#### Standard of care

Key informants noted that establishing the COMFORT CPG as the new standard of care for management of peripartum pain, both regionally and nationally, could help facilitate COMFORT CPG adoption among clinicians. Informants highlighted the importance of CPG endorsement by national professional society endorsements and perinatal quality collaboratives to firmly establish it as the standard of care. Some noted that pressure from performance measurement or value-based reimbursement via OBI’s P4P scorecard could catalyze adoption of CPG-concordant care.

#### Relative priority

Hospital champions noted that maternity units are often working on multiple quality initiatives at once. They described how the demands of competing priorities—for both QI champion teams and frontline clinicians—created initiative fatigue that could impede COMFORT CPG adoption.

#### Structural characteristics

Hospital site champions cited the need to adjust existing local policies and protocols to accommodate the COMFORT CPG recommendations. They also noted how EHR infrastructure could strongly influence CPG adoption. At some sites, EHR changes would necessitate significant time and effort, sometimes requiring a consensus process with several committees, standardization of changes across multiple hospitals within a system, and deferment to other priority projects awaiting EHR changes. At other sites, the ease of implementing a new orderset or standardized documentation phrases was a perceived facilitator to promoting widespread practice change. Additionally, some clinicians noted that their hospital pharmacies carried only combined opioid-acetaminophen formulations and would need to begin stocking oxycodone to be able to provide COMFORT-concordant care. Relatedly, many adjunctive non-pharmacologic therapies were currently unavailable in hospitals. Clinicians and hospital champions described how offering options such as aromatherapy and mindfulness would be limited by funding and staffing, particularly among smaller hospitals or those with resource limitations.

#### Workflow compatibility

Key informants described time constraints and competing clinical priorities as significant barriers to CPG-recommended patient education and counseling. They described how existing workflows both in prenatal care and during the childbirth hospitalization provided limited time to execute robust shared decision-making in developing peripartum pain management plans—particularly in the context of high patient volumes and need to address chronic medical conditions and/or obstetric complications. Many key informants described how lack of clarity about tasks and responsibilities (e.g., who was responsible for peripartum pain management education and documentation) could impede CPG uptake. Some expressed that administering scheduled acetaminophen and non-steroidal anti-inflammatory drugs (NSAIDs) could be burdensome to nurses if patients decline scheduled medications, while others noted benefits to scheduled dosing, including minimizing clinician time addressing unmanaged pain and mitigating individual biases that may affect whether a patient receives pain medication. Several key informants noted potential resistance to CPG-concordant opioid prescribing ranges driven by anticipated difficulties accommodating phone calls and return clinic visits for uncontrolled postpartum pain. Finally, some described the need for workflow processes that promote communication across inpatient and outpatient care teams in the prenatal, intrapartum, and postpartum periods to facilitate coordination of pain management care.

#### Culture of learning

Key informants noted how unit norms and processes related to learning could affect CPG adoption. Some described a unit with high agility, where clinicians believed they would often change practice as evidence evolved and where the organization had well-established processes and expertise to promote clinician learning. Key informants described how this organizational openness to change would facilitate COMFORT CPG adoption. Others described units where clinicians prized time-honored ways of practicing medicine and a general resistance to clinical practice evolution.

#### QI Team characteristics

Key informants described the need for skilled, effective teams to lead implementation of the COMFORT CPG. They cited the importance of champions being persuasive communicators who could convey the rationale for the QI initiative and the evidence base underlying COMFORT CPG recommendations. They highlighted the benefits of multidisciplinary teams, as they could more effectively engage different subgroups on the unit, and the need for clear delineation of the roles and responsibilities of each team member. They also underscored the importance of having a broad coalition leading the initiative and promoting its goals during daily clinical care delivery.

#### Individual provider characteristics

Many hospital champions described perceiving the CPG’s recommendation for scheduled non-opioid medications after cesarean and vaginal birth as relatively easy to implement. Many hospitals noted they already have these protocols for cesarean birth, and many clinicians have already witnessed benefits of this approach with other opioid-sparing recommendations. The opioid prescribing ranges were welcomed by many key informants as an opportunity to promote opioid stewardship and still personalize management to individual patient needs. Conversely, some anticipated encountering resistance if the CPG was perceived as telling providers how to practice or constraining their clinical judgment for patients with unique medical needs. In particular, some informants described the need to justify the CPG’s recommended opioid prescribing ranges to facilitate CPG uptake. Provider knowledge gaps that could impede CPG adoption, including limited provider knowledge about naloxone prescribing and non-pharmacologic adjunctive therapies (e.g., mindfulness, deep breathing, aromatherapy, acupuncture); how to counsel their patients on use of these therapies; and where to direct patients for more information were also described as potential challenges. Finally, some noted that not all attending physicians and midwives were comfortable managing patients with complex pain needs (e.g., chronic pain, history of opioid use disorder) and that only some sites had access to addiction medicine specialists, which could impede efforts to create coordinated pain management plans as recommended by the CPG for populations with complex pain management needs.

#### Individual nurse characteristics

Key informants emphasized the critical role of nurses in pain management and the influence of nurses’ beliefs on CPG adoption. Hospital QI champions described how clinical experiences at the bedside often shaped nurses’ beliefs about postpartum pain management, acceptability of scheduled acetaminophen and NSAIDs, and preferences for staggered versus simultaneous dosing. They noted how nurses’ ability to observe better outcomes with CPG-concordant care could be a strong facilitator of adoption. Resistance to scheduled non-opioid medications was anticipated by some hospital champions due to potential added work of administering scheduled medications and beliefs that patients should be in sufficient pain to request intervention before dosing medication. They noted how emphasizing the medical literature and evidence base for the COMFORT CPG could promote adoption.

#### Individual patient characteristics

Patients identified a strong unmet need for anticipatory counseling about postpartum pain management. They noted large variability in the extent and timing of pain management counseling received during prenatal visits and hospital admissions, leading to inconsistent expectations for some patients about pain experiences following discharge. Many patients described a strong desire to be active participants in developing their pain management care plans, voicing a desire for improved shared decision-making in discussions with clinicians. Additionally, many described a desire for improved continuity of pain management care across prenatal, inpatient, and postpartum settings, and the need to adjust care plans to the realities of patients’ lives. Some key informants anticipated that birthing people’s receptivity to scheduled acetaminophen and NSAIDs and to opioid prescribing ranges may differ based on their prior birth experiences. Some anticipated transportation barriers and financial constraints (e.g., tradeoffs between purchasing medications and newborn supplies) that could impede opioid and non-opioid medication access after discharge. Key informants also described how implicit biases influence opioid dosing and provision of non-medication therapies and described how patients might welcome the CPG as a way to standardize peripartum pain management counseling and offerings and thereby promote care that better meets patients’ needs.

### Implementation intervention for perinatal quality collaboratives

Table 4 presents an implementation intervention for use by quality collaboratives’ coordinating teams to support unit-based champion teams in their work to implement the COMFORT CPG. *Manualization of CPG*: We “packaged” the CPG into a Clinical Practice Guide, which utilizes visually pleasing elements (e.g., images, white space, call-out boxes) and quotes from this qualitative work to emphasize key messages and to encourage engagement by busy clinicians. We also translated the CPG into a sample unit protocol to assist unit-based teams in embedding recommended clinical practices into routine care delivery. *Clinician training*: We offered a 60-min live webinar on two occasions, with a recording available for asynchronous viewing, to create standardized, high-quality, scalable educational content about the CPG for all clinicians at OBI hospitals. Content focused on addressing clinician knowledge gaps anticipated by key informants, especially related to opioid-sparing regimens; population- and procedure-specific opioid prescribing ranges; considerations for patients with a history of pain or substance use disorder; non-medication pain management strategies; and opportunities to promote more equitable and respectful pain management care. *Technical assistance*: Based on our qualitative findings, we identified 12 tools (three identified by participants and an additional nine identified by the study team) that quality collaboratives can disseminate to help unit-based teams optimize local conditions for CPG adoption, which we created and made publicly available on OBI’s website (obstetricsinitiative.org). Table 5 describes each tool’s goal, barriers addressed, and potential uses. Additionally, OBI Outreach Coordinators are available to answer unit-based teams’ questions by email. *Performance feedback*: With end-user input via our focus groups, we designed a push report to help member hospitals track trends in opioid-sparing postpartum pain management and opioid prescribing at delivery hospitalization discharge by birth type (e.g., vaginal birth without complications, vaginal birth with advanced laceration, cesarean birth). Reports were initially distributed quarterly, with an increase in cadence to weekly in the second quarter of 2024. *Facilitation*: Based on end-user input about optimizing both feasibility and effectiveness, we offered two virtual meetings per month for six months, with one conducted in person if desired by a site, and moving to one virtual meeting per month if performance improves—up to a total of 10 30–60 min sessions over six months. *P4P metric*: We designed a P4P metric focused on promoting opioid-sparing postpartum pain management and opioid prescribing within COMFORT CPG ranges.

**Table 4:** Specification of Collaborative-Level Implementation Intervention

Domain	Intervention Component					
	**REP**				**Enhanced REP**	**P4P**
	**Manualization of CPG**	**Clinician Training**	**Technical Assistance**	**Performance Feedback**	**Add Facilitation**	**P4P Metric**
Actor	OBI Coordinating Center	OBI Coordinating Center	OBI Coordinating Center	OBI Coordinating Center	OBI Coordinating Center	Payer
Action	Package CPG recommendations in an accessible, inviting visual document	Training of QI champion teams and frontline clinicians in CPG	Website with QI resources to support the effectiveness of site QI champions	Provide precision feedback on prescribing performance at the hospital and individual clinician level, compared to peers in OBI	Facilitator in OBI Coordinating Center meets with unit-based teams responsible for implementing COMFORT CPG for 1) Relationship-building, 2) Problem-solving, 3) Ongoing monitoring, 4) Planning for sustainment	Provide increased pay for CPG-concordant performance
Target	Hospital QI Champion Team	Hospital QI Champion Team	Hospital QI Champion Team	Hospital QI Champion Team	Hospital QI Champion Team	Hospital
Temporality	Disseminated Fourth Quarter of Fiscal Year 2023 (4Q23) and available thereafter	Offered virtually in 4Q23 and First Quarter- FY2024 and recording available thereafter	Resources disseminated 4Q23 and available thereafter OBI Coordinating Center staff available by email and phone continuously throughout	Push reports (opioid prescribing rate and amount at discharge; inpatient acetaminophen/NSAID ordering) disseminated starting quarter 4, 2023, quarter 1 2024, and then weekly throughout	Offer two virtual meetings per month for 6 months. Up to one conducted in person. Once identified, measure improves, move to 1 virtual call per month	Annual payment based on performance in the prior year
Dose	Document publicly available on an ongoing basis online	Single 60-min webinar/recording	Variable as needed: Ongoing	Ongoing	Up to 10: 30–60-min virtual sessions in 6 months	Variable based on number of births and number of points achieved
Implementation outcome(s) affected	Adoption Fidelity Penetration Reach Effectiveness	Adoption Fidelity Penetration Reach Effectiveness	Feasibility, Acceptability, and Appropriateness of implementation strategies and tools	Adoption Fidelity Penetration Reach Effectiveness	Feasibility, Acceptability, and Appropriateness of facilitation, implementation strategies, and tools Adoption Fidelity Penetration Sustainability	Adoption Fidelity Penetration Reach
Justification	Increase frontline clinicians' knowledge, skills, positive beliefs, and motivation related to the COMFORT CPG [51, 57]	Increase frontline clinicians' knowledge, skills, positive beliefs, and motivation related to the COMFORT CPG [58]	Low cost, highly efficient approach to support multiple hospitals at once through scalable resources and low intensity, centralized consultation with OBI staff [59]	Implicitly regulate clinician behavior by providing data on performance compared to professional standard, target, or peers [60]	Prepare unit-based champions for success by promoting goal- and priority setting, engaging leaders, clarifying roles and responsibilities, coalition-building, problem-solving and strategic thinking, adapting clinical processes, managing team processes, address resistance, and accountability for change [61]	Increase hospital motivation to prioritize and devote needed resources to the initiative [62–65]
Tool	Clinician practice guide Sample unit protocol	COMFORT CPG webinar recording	Champion roles/responsibilities C-suite letter Unit assessment tool Strategies checklist QI blueprint FAQ Protocol template EHR template Patient educational materials	OBI registry dashboard OBI hospital performance report	Facilitation Manual	OBI P4P scorecard metric

*REP* Replicating Effective Programs, *P4P* pay for performance, *CPG* clinical practice guideline, *OBI* Obstetrics Initiative, *QI* quality improvement, *FAQ* frequently asked questions

**Table 5:** Quality Improvement Tools and Goals, Barrier Addressed, and Potential Uses

Tool	Goal of Tool	Barrier Addressed	Potential Uses of Tool
Champion Roles/Responsibilities document	Create shared understanding of roles and responsibilities of unit-based QI champion team members roles and responsibilities	QI leadership	QI teams can utilize to promote coordinated, effective team efforts
C-suite letter	Promote hospital leadership's awareness, buy-in, and engagement in the QI effort	Hospital leader engagement	Distribute to hospital and/or healthcare system leaders to help secure visible support and needed resources
Hospital performance report	Create shared understanding of and motivation to optimize performance	Provider and Nurse Characteristics	Distribute to clinicians and use to scaffold dialogue about the initiative's rationale and clinical significance Use at regular intervals to keep clinicians aware of progress and motivated to achieve and sustain desired performance
Unit self-assessment tool	Identify local barriers and facilitators to clinical practice change	QI leadership	Unit-based team utilizes to create a plan for clinical practice change that meets local needs
Checklist of core strategies	Offer theory-based improvement interventions to assist unit-based teams in planning their QI initiative	QI leadership	Unit-based team selects strategies and tools that support an improvement plan that meets local needs
Bringing Our Patients COMFORT: QI Blueprint	Prompt QI teams to outline a QI plan for implementing COMFORT practices	QI leadership	Unit-based team updates QI plan at monthly meetings, to create shared understanding about initiative goals, progress, and action items and their owners and deadlines
FAQ document	Answer common questions about the COMFORT CPG and its implementation	QI leadership	Share with QI team leaders, or all unit clinicians, to address common knowledge gaps
COMFORT CPG-concordant protocol template	Document unit expectations for CPG-recommended clinical behaviors	Standard of Care, Structural Characteristics	Edit and adopt unit-based pain management processes and protocols based on COMFORT CPG's recommended practices
COMFORT Electronic Health Record (EHR) Templates	Standardize patient record-keeping processes and streamline clinician workflow and time needed for documentation	Workflow Compatibility, Individual Provider and Nurse characteristics	Add standard ordersets to direct post-birth pain management and discharge prescribing; Add standard documentation phrases; Add best practices alert to promote opioid prescribing within COMFORT ranges
Clinician Practice Guide	Provide clinicians with comprehensive, easy-to-read overview of COMFORT practices	Provider and Nurse Characteristics	Distribute to frontline clinicians
Patient Educational Materials	Provide patients with easy-to-understand information about COMFORT practices and their benefits, thereby enhancing patient understanding of recommended care practices and empowerment to take an active role in their care	Individual Patient Characteristics	Distribute to birthing people during third trimester prenatal care; Distribute to birthing people on the labor and delivery unit

*QI* quality improvement, *CPG* clinical practice guideline, *FAQ* frequently asked questions

### Implementation strategies and tools for hospital-based quality improvement teams

**Fig. 2:**
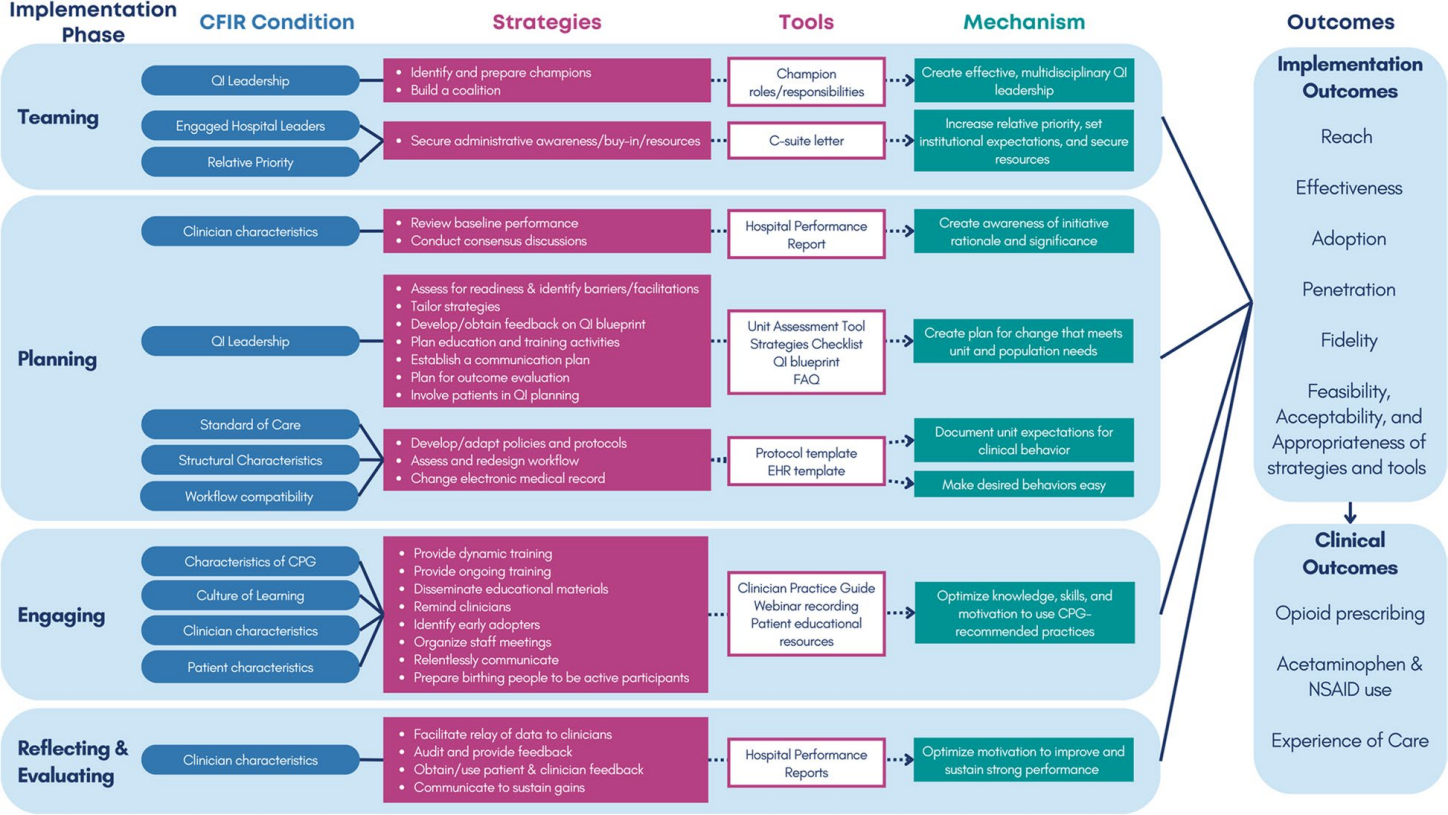
Logic Model

We identified 27 strategies (nine explicitly identified by interview and focus group participants and an additional 18 emerging from the study team’s manual comparison) with 12 associated tools for unit-based QI teams to utilize to optimize identified CFIR conditions (Fig. 2).

#### Teaming

Key strategies included identifying and preparing champions (corresponding tool: document clarifying champion roles and responsibilities); building a coalition; and securing hospital and unit leaders’ buy-in for adopting the COMFORT CPG (tool: C-suite letter to engage organizational leaders).

#### Planning

Reviewing baseline performance (tool: hospital performance report) to identify improvement opportunities and conducting consensus-building dialogue with key constituencies were important strategies to create a shared understanding of the initiative’s rationale and clinical significance. Multiple planning strategies emerged as crucial to help unit-based champion teams create an implementation plan responsive to local clinician and patient needs (tools: unit self-assessment tool to measure barriers/facilitators, checklist of core strategies, QI blueprint to document the site’s implementation plan, FAQ document to address common unit-based champion questions). Participants described the importance of adapting unit policies/protocols (tool: Sample CPG-concordant protocol) and EHR elements (tool: template orderset and counseling documentation) to communicate unit expectations for CPG-concordant clinical behaviors and make desired behaviors easy for clinicians to execute.

#### Engaging

Strategies for providing dynamic, multidisciplinary, and ongoing clinician training; just-in-time reminders; and consistent and repeated communication emerged as likely essential for optimizing clinician perceptions of the CPG and knowledge, skills, capacity, and motivation to use CPG-recommended practices (tools: clinician practice guide, on-demand viewable webinar recording). Communications that focus on the initiative’s priority and the CPG’s evidence base and potential to improve clinical outcomes, the patient experience, and healthcare inequities may be most successful at engaging clinician groups. Elevating the experiences of early adopters and organizing meetings with frontline clinicians emerged as important strategies to demonstrate that desired changes are possible and beneficial and to foster momentum and self-efficacy among clinicians trying new practices. Patient educational materials emerged as key resources for helping to prepare birthing people to be active participants in developing their pain management care plans and knowledge and motivation to accept CPG recommendations.

#### Reflecting & evaluating

Key data-driven strategies to motivate consistent improvement efforts and sustained strong performance included conveying unit-based data to frontline clinicians, auditing and providing feedback to individual clinicians, obtaining and sharing patient and clinician feedback about the initiative, and ongoing communication about initiative progress and performance outcomes (tool: hospital performance report).

## Discussion

This study characterizes key contextual conditions likely to influence implementation of the COMFORT CPG and specifies corresponding theory-based implementation strategies and associated tools for unit-based QI teams and statewide quality collaboratives to help embed the COMFORT CPG into routine care delivery across the United States.

The influential conditions identified align with a 2018 systematic review of barriers and facilitators to hospital-based implementation processes, which identified environmental context (e.g., workflow, competing demands, staffing); culture; individual clinician factors (e.g., staff commitment/attitudes and skills/knowledge/confidence); and ease of intervention integration as frequently cited determinants of implementation outcomes across 43 studies [66]. Our study further advances this work by characterizing these contextual factors within contemporary, diverse maternity settings and by linking each determinant to strategies with plausible mechanisms of effect on implementation and clinical outcomes. Our work also builds on a 2006 systematic review of implementation strategies for clinical practice guidelines in obstetric care, which identified reminders, audit and feedback, and multifaceted interventions as key change strategies and underscored the importance of aligning strategies with identified local barriers to practice change [67]. Our findings advance this work by elucidating hypothesized mechanisms by which strategies might act on contextual factors (i.e., mitigate barriers and leverage facilitators) to promote successful implementation.

Formative, theory-informed work to design improvement interventions is limited in the field of maternity care QI [68]. Our study is novel in its use of a systematic process, based on well-established theoretical frameworks, to design implementation interventions to promote adoption of newly recommended clinical practices on maternity units. Our linkage of contextual factors to corresponding strategies and tools and hypothesized mechanisms of action may assist unit-based teams in purposefully selecting strategies to optimize local context for CPG adoption. Unit-based QI champion teams can utilize our logic model, self-assessment tool, and QI blueprint to identify local barriers and facilitators and intentionally select corresponding strategies and tools with clear rationale for their use. Our study finds that 27 core strategies may be important, suggesting that unit-based teams will need to be appropriately resourced to successfully adopt the COMFORT CPG.

Our findings can also be used by perinatal quality collaboratives to support unit-based teams in their local work to drive CPG adoption. A key strength is that the collaborative-level implementation intervention is now based on end-user input. Moreover, it is specified in sufficient detail that it can be prospectively evaluated at the statewide level to elucidate how statewide collaboratives can best support hospital members (i.e., what types of support work for which types of hospitals, under what contextual conditions, and via which mechanisms on implementation and clinical outcomes). The collaborative-level strategies and associated tools may require modification as they are utilized prospectively in real-world settings. For example, our research team decided to utilize a didactic, webinar format for provider training to maximize efficient scalability; however, as Vamos et al. demonstrate in their recent scoping review [58], a number of training techniques are available (e.g., simulations, hands-on education like scenario, role play, or demonstration, and discussion) and combining active learning techniques may ultimately be the most effective at promoting the sustained behavior change required for COMFORT CPG adoption [69–73].

Researchers can utilize our findings to advance the evidence base for maternity QI. For example, researchers can prospectively measure which strategies unit-based QI teams choose to utilize and whether they address both barriers and facilitators to COMFORT CPG adoption and downstream implementation and clinical outcomes, as hypothesized herein.

Our findings should be interpreted within our study’s limitations. First, our pragmatic design leveraged the research team’s expertise to select ERIC strategies but did not allow for unit-based QI teams to quantitatively rank and prioritize ERIC strategies (e.g., based on perceived importance or feasibility at their site); we hope to refine our list of core strategies based on retrospective reflection and discussion with unit-based QI teams after COMFORT CPG implementation in OBI. Second, our QI champions were all working within hospitals in Michigan; the implementation barriers and facilitators in Michigan hospitals may not perfectly generalize to other settings. However, the heterogeneity of care settings represented by our participant hospitals strengthens the external validity of our findings. Third, as with any study based on self-report, our findings are subject to social desirability bias. Additionally, individuals choosing to participate in focus groups may be more engaged in OBI and hold more favorable views of the COMFORT CPG, or QI more broadly, than those not volunteering to participate. Finally, the implementation interventions generated were informed by anticipated barriers identified by key informants prior to implementation; additional barriers and needed strategies and tools may emerge during real-world implementation. These limitations notwithstanding, key study strengths include a robust theoretical basis for study methodologies; the involvement of a national and statewide sample of birthing people, clinicians, and QI leaders as “end users” to inform the design of implementation interventions; and the triangulation of observations across these multiple types of key informants to enhance internal validity of our findings.

## Conclusion

Overall, this study is novel in the use of well-established theoretical frameworks (CFIR, ERIC, E-REP) to identify key influential conditions for implementation of a specific CPG, the COMFORT CPG for postpartum pain management; develop a toolkit of ERIC strategies for unit-based QI teams; and propose an implementation package for perinatal quality collaboratives to support multidisciplinary QI teams informed by E-REP. Prospective research is now needed to evaluate the COMFORT CPG as implemented in real-world settings using the strategies and resources created herein.

## Data Availability

The datasets used and/or analyzed during the current study are available from the corresponding author on reasonable request.

## Abbreviations

COMFORT: Creating Optimal pain Management FOR Tailoring care
CPG: Clinical practice guideline
EHR: Electronic health record
QI: Quality improvement
NSAIDs: Non-steroidal anti-inflammatory drugs
OBI: Obstetrics Initiative
REP: Replicating Effective Programs
E-REP: Enhanced Replicating Effective Programs
CFIR: Consolidated Framework for Implementation Research
ERIC: Expert Recommendations for Implementing Change
